# The Influence of Wing Membrane Elasticity on Aerodynamics in a Bat-Inspired Flapping Robot

**DOI:** 10.3390/biomimetics10030161

**Published:** 2025-03-05

**Authors:** Szu-I Yeh, Chia-Hsu Chiang

**Affiliations:** Department of Aeronautics and Astronautics Engineering, National Cheng Kung University, Tainan City 701, Taiwan

**Keywords:** bat-inspired flapping robot, wing membrane elasticity, aerodynamic force, unsteady aerodynamics, folding mechanism

## Abstract

This study investigates the aerodynamic effects of wing membrane elasticity inspired by bats, which exhibit exceptional maneuverability and stability. By mimicking bat wing folding and flapping motions, a 2-DOF flapping mechanism was developed to examine the impact of wing membrane elasticity. Polydimethylsiloxane (PDMS) membranes with tunable elastic properties were fabricated by adjusting the ratio of the curing agent (B agent), with the 1/50 ratio exhibiting the greatest extensibility and the lowest Young’s modulus. Experimental results demonstrate that wing membrane elasticity significantly influences aerodynamic performance. During flapping, increased elasticity led to larger camber changes, enhancing vertical lift through stronger leading-edge vortices, as confirmed by PIV flow field measurements. However, when elasticity became excessively high, as in the 1/50 membrane, the lift benefit diminished, and horizontal force decreased, indicating a trade-off between vertical and horizontal aerodynamic performance. Additionally, the folding mechanism was found to be critical for drag reduction, reducing nearly 50% of negative horizontal forces during flight. By integrating adjustable wing membrane properties and a bioinspired flapping mechanism, this research provides valuable insights into the aerodynamic characteristics of bat flight. These findings not only enhance the understanding of flapping wing aerodynamics but also offer guidance for the design of efficient and agile bioinspired aerial vehicles.

## 1. Introduction

In the development of the aviation field, bioinspired flight has emerged as a fascinating area of research. By observing the flight mechanisms and structures of various organisms in nature, researchers have uncovered numerous elegant and efficient flight systems, such as the wings of birds, the membranous wings of insects, and the patagial wings of bats [1,2]. These unique wing structures allow these organisms to fly freely in open spaces and maneuver with remarkable precision in confined environments.

In nature, birds rely on forelimbs supported by skeletal structures, enabling a high degree of freedom (DOF) at various joints during flight. For example, coordinated movements of the shoulder and elbow joints reduce wing surface area during the upstroke [3], minimizing negative lift, while the alula, controlled by the thumb, helps stabilize the leading-edge vortex (LEV) to maintain flight performance [4]. These adaptations significantly enhance aerodynamic efficiency. Insects, on the other hand, use membranous wings powered by high-frequency flapping, where the elasticity of the wing membranes affects both aerodynamic and inertial forces [5,6]. Bats, as the only mammals capable of powered flight, possess a distinctive wing structure primarily composed of flexible membranes (patagia) connected to their limb skeletons. By employing muscular tension and skeletal control, bats can adjust the tension of their wing membranes to alter wing shape and area, adapting to various flight demands. This unique combination of traits found in birds and insects enables bats to perform complex flight maneuvers and leverage the elasticity of their wing membranes [7,8]. These characteristics not only enhance their flight performance but also present valuable opportunities for advancing bioinspired flight research.

Bats can adjust the tension of their wing membranes through muscular control, enabling them to modulate wing camber and adapt to varying flight conditions. Research has investigated the role of membrane tension in its performance in flight. For example, electromyographic (EMG) measurements have demonstrated that the lateral wing membrane muscles remain active during the downstroke, sustaining tension in the wing membrane. This tension is critical for maintaining flight stability and preventing excessive deformation during flapping [9]. In another study, researchers used botulinum toxin to temporarily paralyze the muscles controlling the lateral wing membrane, reducing the ability to maintain tension. This resulted in a noticeable increase in wing camber, which led to higher aerodynamic drag and decreased flight speed. The study also found that bats were unable to sustain flight under certain conditions without sufficient membrane tension. To mitigate the negative effects of increased drag, the bats adjusted their flapping motion by altering stroke plane angles and flapping amplitudes, highlighting the dynamic interaction between wing membrane tension and flight mechanics [10].

Beyond biological studies, research on flexible flapping wings has further demonstrated the aerodynamic significance of wing compliance. Studies on the clap-and-fling mechanism have shown that wing flexibility modifies this lift-enhancing strategy by transforming it into a clap-and-peel motion, which increases circulation during the fling phase and reduces drag by enabling passive deformation under aerodynamic loads [11]. Additionally, investigations using a simplified hawkmoth-inspired model have revealed that moderate flexibility stabilizes the leading-edge vortex and enhances lift generation, whereas excessive flexibility can lead to vortex instability and reduced aerodynamic efficiency [12]. Another study introduced the Slack Angle to quantify structural flexibility in flapping wings, analyzing its impact on aerodynamic force production and vortex dynamics. The experiments compared PET membrane wings with rigid wings, showing that flexible wings generate larger tip vortices, which contribute to improved aerodynamic efficiency in flapping-wing systems [13]. Over the past decade, several review papers have systematically examined the aerodynamic effects of flexible wing surfaces under flapping conditions. Recent review papers [14,15] have examined the aerodynamic effects of flexible wing structures in flapping-wing systems, primarily from a fluid dynamics perspective. While various studies have explored the influence of material compliance on vortex formation and force generation, most analyses rely on simplified kinematics and structural approximations to facilitate controlled experiments and numerical modeling. However, due to the wide range of scales and diverse kinematic patterns observed in biological flapping flight, existing studies have yet to establish a comprehensive understanding that accounts for these variations, leaving many aspects open for further investigation. Understanding how wing flexibility interacts with different flight modes, vortex dynamics, and aerodynamic force generation continues to be a crucial area of research, particularly in developing bioinspired flapping-wing systems.

In the study of aerodynamic effects caused by wing membrane deformation, replicating the complex flight motions of bats remains challenging due to their dynamic wing area adjustments during folding and camber changes driven by muscle tension variations. To simplify experimental conditions, D. K. Riskin et al. quantified bat flight kinematics, while K. Viswanath et al. used motion analysis and numerical simulations to reconstruct bat flight and analyze flow fields and aerodynamic forces [16,17]. Building on these efforts, Y. Yu and Z. Guan. modeled four key types of bat wing deformations: torsional deformations along the span, camber changes controlled by finger joints, bending during the upstroke, and wing area variations caused by joint movements. These models demonstrated improved lift compared to rigid wings, with camber and wing area variations showing the most significant aerodynamic benefits [18]. Additionally, research into the mechanical properties of membrane materials has led to more realistic wing designs by incorporating materials with varying elasticity and vein structures, further advancing bioinspired flapping wing aerodynamics [5].

Based on the reviewed literature, it is evident that bats exhibit highly complex wing movements during flight, driven by the coordinated motions of the elbow, wrist, and finger joints. Their elastic wing membranes not only maintain tension and provide protection but also play a critical role in adapting to various flight conditions. The combination of these features results in intricate wing deformations that cannot be overlooked when studying bat flight aerodynamics. Due to the complexity of these movements, most existing research relies on numerical simulations. Although a few multi-DOF robots with folding mechanisms have been developed [19,20,21,22], they primarily focus on mechanical design and control systems rather than aerodynamic performance under combined motions. Additionally, the influence of wing membrane properties, including material elasticity, remains underexplored despite its critical impact on aerodynamic behavior. This study aims to address these gaps by investigating the effects of wing membrane elasticity on aerodynamic forces and flow fields during hovering flight, using a bat-inspired flapping robot with 2-DOFs to replicate wing folding and flapping motions. Wing membranes with adjustable mechanical properties will be fabricated, and experiments will be conducted to measure aerodynamic performance and analyze flow structures. The findings will not only advance the understanding of bat flight characteristics but also provide valuable insights for designing more efficient and agile bioinspired aerial vehicles.

## 2. Research Methodology

### 2.1. Flapping Mechanism Design

The bioinspired flapping mechanism in this study is based on the lesser long-tongued bat (*Leptonycteris yerbabuenae*), a species commonly found in Central and South America. This bat inhabits semi-arid grasslands, shrublands, and forests, requiring high maneuverability for navigation in its environment. Notably, it possesses strong hovering capabilities, making it an ideal reference for this study. Additionally, this species exhibits seasonal migration, demonstrating the ability to switch between various flight modes, which provides a valuable foundation for further studies on its flight performance.

The morphological parameters of the lesser long-nosed bat were adopted from previous research [23]. The reference species has a semi-wingspan of 167.5 mm, and given the experimental constraints, the flapping mechanism in this study was designed with a wingspan of 200 mm, resulting in a scaling factor of 1.194 relative to the biological counterpart. While the model was developed to resemble the biological morphology as closely as possible, practical constraints related to mechanical actuation and wing deformation control necessitated minor modifications. Such adjustments are common in bioinspired aerodynamic studies to ensure functionality while maintaining key aerodynamic characteristics. Differences within the rounding range of the same order of magnitude do not significantly affect the experimental outcomes. Since the experiments were conducted in a water tank, the wall effect on the flow field needed to be minimized. Previous studies on flapping mechanisms have suggested that the distance between the wingtip and the tank wall should be at least three times the mean chord length to neglect wall-induced disturbances [24]. The water tank used in this study had a short side length of 800 mm, ensuring that the wingtip-to-wall distance was 200 mm, which satisfies the condition to minimize wall interference.

To maintain dynamic similarity, the Reynolds number (*Re*) and Strouhal number (*St*) were matched to those of a hovering bat. As no direct reference values for wingtip velocity were available, an estimate was derived using semi-wingspan, stroke amplitude, and flapping frequency. Calculations indicated that the real bat’s average wingtip velocity ranged from 3.53 to 3.97 m/s, corresponding to a Reynolds number of 10,600–12,000. Based on these non-dimensional parameters, the required wingtip velocity for the experimental setup was determined to be 0.153–0.172 m/s, with a flapping frequency of 0.4–0.51 Hz. These two parameters have been widely recognized in previous studies as the most critical factors for ensuring the validity of water-based flapping wing experiments [25]. The comparative specifications of the real bat and the designed flapping mechanism, along with their corresponding non-dimensional parameters, are summarized in Table 1.

**Figure 1:**
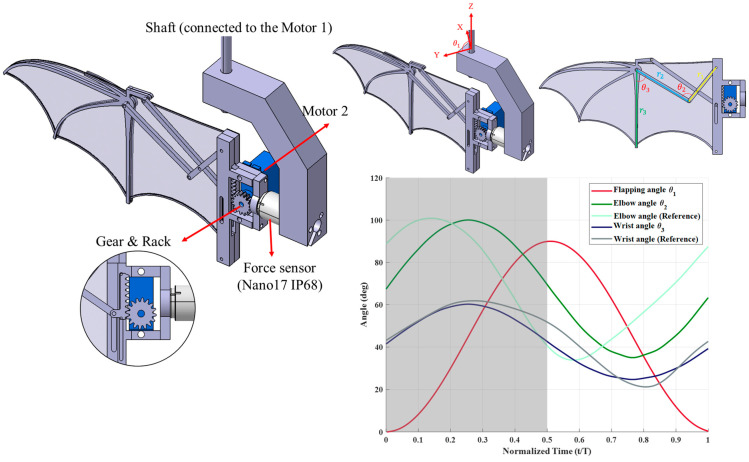
Schematic of the bat-inspired 2-DOF flapping mechanism, showing the flapping and folding motions, angular definitions, and one cycle of angle variations. The gray-shaded regions in the angle variation plot represent the downstroke, while the white regions correspond to the upstroke. To verify whether the actual motion of the mechanism aligns with the prescribed angular trajectories, a Direct Linear Transformation (DLT) method was applied. Three cameras were used to capture the flapping motion, and the 2D coordinates of feature points in the images were transformed into 3D spatial coordinates. By computing the vectors between these points and using an inner product method, the angular variations in the wings were analyzed. The correlation between the measured and prescribed flapping angles indicated that while minor motion delays occurred when using different wing membranes, the correlation coefficient remained around 0.9. Additionally, both flapping amplitude and frequency closely matched the set values, with only minimal deviations. For the folding motion, the four-bar linkage mechanism, driven by a rack-and-pinion system, provided stable power transmission with minimal external disturbances. Across all three wing membrane conditions, the measured elbow and wrist joint angles closely matched the intended trajectories, with correlation coefficients exceeding 0.98, confirming the reliability and stability of the folding motion.

The flapping mechanism designed in this study features two degrees of freedom (2-DOF), incorporating both flapping and folding motions. The structure is fabricated using fused deposition modeling (FDM)3D printing technology, with Polyethylene Terephthalate Glycol (PETG) as the primary material. The flapping motion is driven by Motor 1, which connects to an aluminum-made shaft, while Motor 2 controls the folding motion through a rack-and-pinion system and a four-bar linkage mechanism. Additionally, a force sensor is installed to measure aerodynamic forces generated during the experiments, as shown in Figure 1.

Since the forelimbs of real bats are connected to the wing membrane, natural wing folding significantly alters the inner-wing area. Directly attaching the membrane to the four-bar linkage in the designed mechanism would cause excessive stress, leading to membrane rupture. To address this, the inner-wing section was separated from the linkage. Previous observations on hovering bats [26] revealed that during the upstroke, the inner wing exhibits a cambered, inflated shape. To replicate this feature, a Styrene Block Copolymer (SBC) with a thickness of 0.6 mm was used to secure the wing membrane edges, allowing the inner wing to assume a natural curved profile.

The 2-DOF mechanism was designed based on the kinematics of real bat hovering flight [23,27] incorporating flapping and folding motions to replicate key aspects of bat wing dynamics (Figure 1). According to previous observations, the flapping angle variation closely follows a sinusoidal function, while the folding motion, despite exhibiting more variation during hovering, can be reasonably approximated using a cosine function. Since finger joint angles exhibit minimal changes, only the elbow and wrist joints, which undergo significant movement, were incorporated into the folding mechanism, while the finger joints remained fixed. The mechanism’s motion was symmetrically adjusted compared to real bat kinematics to ensure more stable aerodynamic and inertial force characteristics. This modification helps mitigate sudden aerodynamic and inertial force fluctuations that could arise from abrupt motion changes. Additionally, due to the kinematic constraints of the four-bar linkage system, the elbow and wrist joint motions cannot perfectly match the reference angles obtained from biological studies. Compromising this limitation, the maximum and minimum joint angles reported in the literature were adopted as reference values, allowing for a refined motion profile that balances biomechanical accuracy and mechanical feasibility. These design considerations ensure that the mechanism captures the essential aerodynamic behaviors of bat flight while maintaining a practical and controllable flapping system for experimental analysis.

### 2.2. Wing Membrane Design and Elasticity

**Figure 2:**
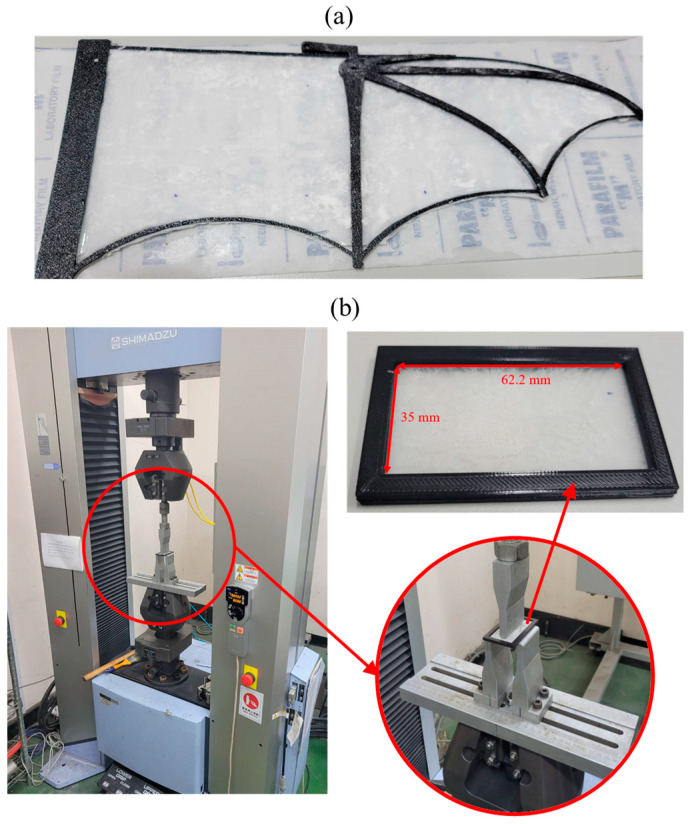
(**a**) Assembled wing membrane used in the experiment. (**b**) Schematic of the three-point bending test setup for measuring the flexural stiffness of wing membranes.

To simulate the elasticity of bat wing membranes, this study utilized polydimethylsiloxane (PDMS) as the membrane material. PDMS was fabricated by mixing a base agent (A) with a curing agent (B), where the ratio of A:B was set to 10:1, 30:1, and 50:1 as experimental parameters. The initial membrane was cast in a 220 mm × 110 mm mold. After curing, a digital caliper with 0.01 mm precision was used to measure the thickness at multiple points to obtain an average value ($\bar{h}$). The membranes were then cut into bat-wing shapes and bonded to a 3D-printed PETG frame for integration into the flapping mechanism, which is shown in Figure 2a. During the curing process, slight height variations led to non-uniform thickness distribution across the membrane. To quantify the thickness consistency, the coefficient of variation (CV) was used, defined as the ratio of the standard deviation to the average thickness. As shown in Table 2, the 10:1 membrane exhibited an average thickness of 0.21 mm with a CV of 4.7%, indicating relatively uniform thickness. Conversely, the 50:1 membrane had the highest CV (16.2%), due to the increased viscosity of PDMS at higher A-agent ratios, making it more challenging to spread evenly. Despite these variations, all three membranes maintained sufficient uniformity for experimental purposes.

The average thickness of all membranes was approximately 0.2 mm, except for the 30:1 membrane, which was slightly thicker at 0.23 mm—about 15% thicker than the other two. Given the thickness differences among the membranes, previous research [28] investigated the aerodynamic effects of camber and membrane wings. This study incorporated membrane thickness corrections into aerodynamic analysis by introducing the Aeroelastic number (*Ae*), a dimensionless parameter representing the ratio of wing membrane compliance to dynamic pressure.

$$Ae=\frac{E\bar{h}}{\frac{1}{2}\rho {\bar{V}}_{\mathrm{tip}}^{2}\bar{c}}$$

The elasticity of the wing membrane was measured using a three-point bending test, which determines the flexural stiffness of the material [29]. The testing setup is illustrated in Figure 2b. The membrane specimen was mounted on a test fixture with a fixed span length (*L*), while a central force (*P*) was applied at the midpoint between two support points. As the applied force increased, the corresponding displacement (*δ*) was recorded, and Young’s modulus (*E*) was calculated using the equation listed below. Since the span length remained constant, the ratio of applied force to displacement was proportional to Young’s modulus, and thus, this ratio was used as a relative measure of membrane stiffness in this study. The dimensions of the test specimens were also designed according to the constraints of the three-point bending test apparatus, as shown in Figure 2b. The specimen length was set to 35 mm, matching the minimum span length of the bending test machine, while the width was 62.2 mm, corresponding to the mean chord length of the experimental wing model. To ensure consistency with the wing fabrication process, a black PETG frame, identical to the one used in the flapping mechanism, was bonded to the membrane.

The material characterization showed that as the ratio of the curing agent (B agent) decreased, both Young’s modulus and the Aeroelastic number (*Ae*) declined, indicating that the wing membrane became progressively more flexible from 10:1 to 50:1. As observed in Table 2, a reduction in the curing agent ratio led to an increase in maximum displacement, with the 50:1 membrane exhibiting twice the extension compared to the 10:1 membrane, demonstrating significantly greater extensibility and flexibility.

$$\frac{dP}{d\delta }=\frac{48E}{L^{3}}$$

### 2.3. Experimental Setup

The experiment was conducted in a water tank to measure aerodynamic forces and visualize the surrounding flow field under hovering flight conditions. The tank had dimensions of 1800 mm (length) × 800 mm (width) × 900 mm (height), and the flapping mechanism was positioned at the center to ensure a uniform flow environment, free from external disturbances. The closed environment of the water tank provided a stable setting for analyzing zero-freestream conditions, which are representative of hovering flight.

The aerodynamic forces generated by the flapping mechanism were measured using a waterproof ATI Nano17 IP68 six-axis force/torque sensor (ATI Industrial Automation, Apex, NC, USA). The sensor signals were amplified by a 9105-C-PS-U-2 signal amplifier (ATI Industrial Automation, Apex, NC, USA) and acquired at a sampling frequency of 100 Hz using an NI USB-6210 data acquisition system (National Instruments, Austin, TX, USA). To ensure reliable force data, each experiment consisted of five flapping cycles per trial. The first and last cycles, where the flow field was still developing or stabilizing, were excluded. The experiment was repeated three times, yielding a total of nine flapping cycles for analysis. Since the force sensor moved with the flapping mechanism, a coordinate transformation was applied to the recorded signals to resolve the forces into vertical and horizontal components. The forces measured in water included both aerodynamic forces and inertial forces. To isolate the aerodynamic contribution, the same flapping experiments were performed in the air to determine the inertial forces. Given that water is approximately 1000 times denser than air, the forces measured in air were significantly smaller than those measured in water. Thus, the airborne force measurements were used as an estimate of the inertial forces, which were subtracted from the total forces measured in water to obtain the aerodynamic forces.

**Figure 3:**
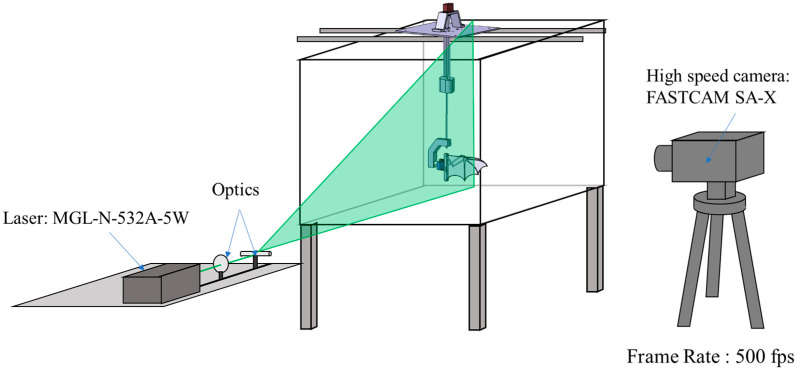
Experimental setup for aerodynamic force measurement and flow field visualization in the water tank.

To observe the effects of the flapping mechanism on the surrounding flow field, Particle Image Velocimetry (PIV) was employed for flow visualization. Hollow glass spheres with a particle diameter of 8–12 µm were dispersed into the water to act as tracer particles. These particles have a density close to that of water, ensuring high flow fidelity and making them well-suited for PIV experiments. A 532 nm MGL-N-532A laser (Changchun New Industries Optoelectronics Technology Co., Ltd., Changchun, China) was used to generate a 1.5 mm thick laser sheet, illuminating the desired observation plane (Figure 3). A FASTCAM SA-X high-speed camera (Photron, Tokyo, Japan) captured images at 500 frames per second (fps). The acquired images were processed using PIVlab 3.09 [30], an open-source software, to extract velocity fields, vorticity fields, and circulation data, with a focus on leading-edge vortex (LEV) dynamics using the Q-criterion.

## 3. Results and Discussion

**Figure 4:**
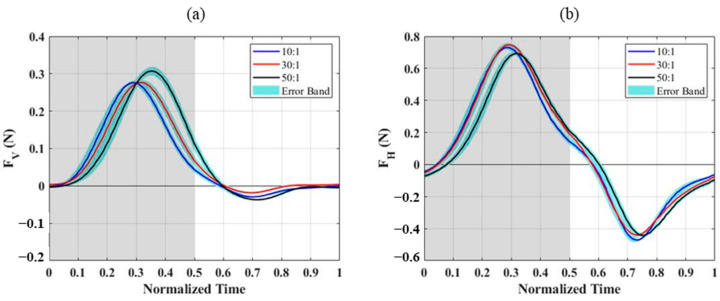
Time variation of vertical and horizontal forces for PDMS wing membranes with different A:B ratios during a flapping cycle: (**a**) vertical force, (**b**) horizontal force. The gray-shaded regions represent the downstroke, while the white regions correspond to the upstroke.

**Figure 5:**
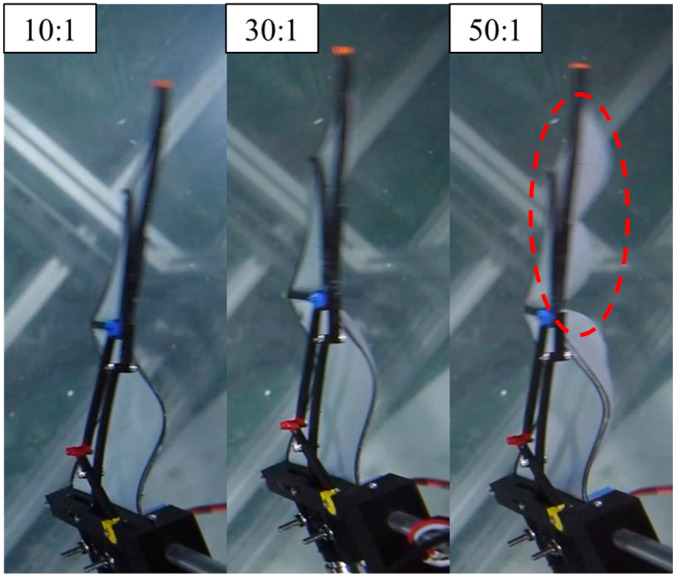
Top-view images showing wing deformation for PDMS wing membranes with different A:B ratios during the downstroke. The 50:1 membrane exhibits the most significant deformation, particularly in the outer wing region, as highlighted in the red marked.

After subtracting the inertial forces obtained from experiments with the wing membrane removed, the aerodynamic forces measured for different PDMS wing membranes are shown in Figure 4. For the 10:1 and 30:1 PDMS wing membranes, the peak vertical and horizontal forces occurred at t/T = 0.3, which corresponds to the phase where the downstroke acceleration is maximal. In contrast, the 50:1 membrane exhibited a delayed peak at t/T = 0.35. This delay was observed in both vertical and horizontal force components, suggesting a difference in aerodynamic response due to the variation in membrane elasticity. From the top-view images (Figure 5), it is evident that the outer wing section of the 50:1 membrane deforms significantly more than those of the other two configurations. This increased deformation is caused by the lower Young’s modulus, which allows the wing membrane to bend more easily under aerodynamic loading, leading to a greater camber formation throughout the flapping cycle. The enhanced camber directly affects the aerodynamic forces generated, contributing to the observed differences in force magnitude and timing among the three wing configurations.

**Figure 6:**
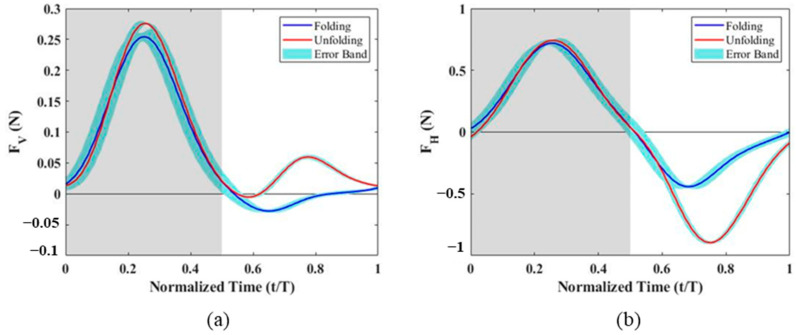
Comparison of aerodynamic forces with and without wing folding for the 50:1 PDMS wing membrane: (**a**) vertical force, (**b**) horizontal force. The gray-shaded regions represent the downstroke, while the white regions correspond to the up-stroke.

Analyzing the upstroke and downstroke forces separately, it was observed that the negative horizontal force during the upstroke was significantly lower, suggesting a strong correlation with wing folding. To further evaluate this effect, additional experiments were conducted without the folding motion, and the results were compared to the original folding configuration (Figure 6). The findings indicate that the vertical force peaks in both upstroke and downstroke were slightly increased when wing folding was disabled. However, the horizontal force during the downstroke remained nearly unchanged. In contrast, during the upstroke, the peak negative horizontal force without folding reached −0.89 N, whereas, with folding, it was only −0.45 N, nearly half of the non-folding value. This suggests that when the wing remains unfolded during the upstroke, the effective wing area increases, leading to a substantial increase in negative horizontal force.

Table 3 summarizes the average aerodynamic forces for the upstroke, downstroke, and the entire flapping cycle. For the vertical force, the average downstroke lift increased as Young’s modulus decreased, indicating that softer membranes generated greater lift. During the upstroke, the 30:1 membrane uniquely exhibited a small positive vertical force, while the other two membranes produced near-zero lift. However, the upstroke-generated vertical force only accounted for 1–5% of the total cycle lift, meaning the overall flapping cycle lift was still dominated by the downstroke force, which increased as the Young’s modulus decreased. For the horizontal force, the upstroke folding motion effectively reduced the negative horizontal force by approximately 50%, as the reduced effective wing area minimized the opposing drag-like force. The average horizontal force over the entire flapping cycle exhibited a nonlinear trend, where the force initially increased and then decreased as the B agent ratio declined. This trend aligns with previous studies [31], which observed that at a fixed angle of attack, as the Aeroelastic number (Ae) decreases, the lift coefficient initially increases but then declines beyond a critical threshold. It is inferred that the 50:1 wing membrane, due to its lower Aeroelastic number, may have exceeded the optimal elasticity range, leading to reduced aerodynamic force generation.

To further validate the aerodynamic trends observed in the force measurements, Particle Image Velocimetry (PIV) experiments were conducted to analyze the leading-edge vortex (LEV) and quantify circulation, providing additional insights into the force variations among different wing membranes. The laser sheet was positioned at 150 mm from the wing root (0.75 wingspan), capturing a plane perpendicular to the wingspan direction. The analysis focused on the mid-downstroke phase (t/T = 0.3) where the maximum vertical force occurred. This phase was selected because aerodynamic loading induces significant wing deformation, leading to camber variations that influence LEV formation.

**Figure 7:**
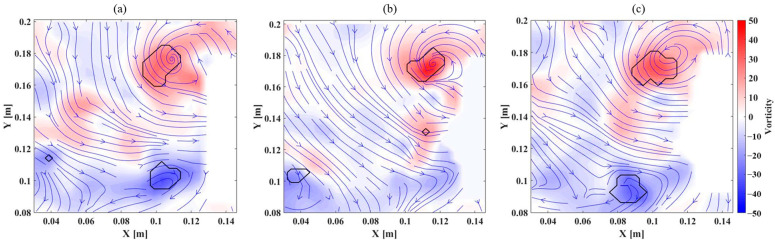
Flow field visualization at 0.75 wingspan during the mid-downstroke (t/T = 0.3) for different PDMS wing membranes: (**a**) 10:1, (**b**) 30:1, (**c**) 50:1.

The flow field distributions for different wing membranes are presented in Figure 7, illustrating how wing flexibility influences vortex structures and jet flow formation during the flapping cycle. For the 10:1 membrane, Figure 7a shows a leading-edge vortex (LEV) and a trailing-edge vortex (TEV) adhering to the wing surface, with a jet flow forming between them. Additionally, a detached TEV appears in the lower-left corner, indicating vortex separation from the wing as it progresses through the flapping cycle. In contrast, the 30:1 membrane, as illustrated in Figure 7b, exhibits a less distinct TEV, which is largely unobservable. This is likely due to the increased camber of the wing membrane, which alters the trailing-edge flow behavior, making it more difficult to track flow particles near this region. Additionally, light reflection from the flexible membrane may have contributed to the difficulty in capturing near-surface flow structures at the trailing edge. However, the LEV and a detached TEV remain identifiable, suggesting that while the overall vortex structure is modified compared to the stiffer membrane, the fundamental aerodynamic interactions persist. For the 50:1 membrane, Figure 7c depicts a larger LEV, indicating that increased flexibility enhances vortex formation at the leading edge. Meanwhile, the TEV shifts further downstream relative to the 10:1 case, suggesting that trailing-edge vortex detachment occurs later in the cycle. Despite this positional shift, the vortex strength remains largely unchanged, implying that the aerodynamic effects generated by the flexible wing are sustained throughout the flapping motion. These observations highlight how membrane flexibility influences vortex dynamics, camber formation, and overall aerodynamic performance, reinforcing the role of structural compliance in modifying unsteady flow characteristics in flapping-wing aerodynamics.

The circulation values computed from the leading-edge vortex further support the observed aerodynamic trends. As the wing membrane stiffness decreases, circulation increases, with values of 0.00811 m²/s for the 10:1 membrane, 0.00941 m²/s for the 30:1 membrane, and 0.01070 m²/s for the 50:1 membrane. These results suggest a strong correlation between camber and vortex formation, where increased camber leads to higher pressure differences at the leading edge, generating a stronger LEV and subsequently increasing circulation and vertical force production. This aligns well with previous studies [32] and is consistent with the vertical force measurements discussed earlier.

## 4. Conclusions

This study developed a bat-inspired 2-DOF flapping mechanism that captures essential wing folding and flapping motions, while simplifying the highly complex multi-joint movements found in real bats. The folding mechanism, powered by a four-bar linkage, enabled stable and repeatable control of the elbow and wrist joint angles, ensuring reliable motion transmission. The integration of 3D printing technology enhanced design adaptability, while self-fabricated PDMS membranes with adjustable elasticity served as aerodynamic surfaces, allowing systematic investigation of wing flexibility effects on aerodynamic performance.

To analyze the impact of wing membrane elasticity, PDMS membranes were fabricated with three different curing agent (B agent) to base material (A agent) ratios (10:1, 30:1, and 50:1), with the 50:1 ratio exhibiting the lowest Young’s modulus and highest extensibility. Experimental results demonstrated that increased elasticity promoted greater camber variation and strengthened leading-edge vortices, leading to enhanced vertical lift generation. However, excessive flexibility, as seen in the 50:1 membrane, resulted in a reduction in horizontal force, which negatively affected overall aerodynamic efficiency. Additionally, the folding mechanism played a crucial role in drag reduction, lowering negative horizontal forces by nearly 50%, highlighting its importance in flapping wing aerodynamics.

These findings provide valuable insights into the role of wing elasticity in flapping-wing aerodynamics, demonstrating that membrane flexibility can significantly influence force production and vortex dynamics. By integrating adjustable wing properties with a bioinspired flapping mechanism, the findings not only advance the understanding of bat flight mechanics but also contribute to the development of more efficient and agile bioinspired aerial vehicles.

## Figures and Tables

**Table 1:** Comparison of the real bat and the designed flapping mechanism in dimensional and non-dimensional parameters.

Parameter	*Leptonycteris yerbabuenae* [23]	Flapping Mechanism
Wing span, *b*	167.5 mm	200 mm
Amplitude, *A*	45–47.5°	45°
Mean chord, $\bar{c}$	47 mm	62.2 mm
Aspect ratio, *AR*	3.56	3.21
Wingtip speed, *V_tip_*	3.53–3.97 m/s	0.15 m/s
Frequency, *f*	13.4–14.3 Hz	0.4 Hz
Reynolds number, *Re*	10,600–12,000	10,680
Strouhal number, *St*	0.159–0.185	0.166

**Table 2:** Thickness and elasticity-related material properties of wing membranes with different curing agent (B agent) ratios.

Parameter	10:1	30:1	50:1
$\bar{h}$ (mm)	0.21	0.23	0.20
CV (%)	4.7	10	16.2
*E* (N/mm^2^)	735	406	296
*Ae*	22.36	13.72	8.85
$\delta_{\max}$ (mm)	12.15	17.44	25.67

**Table 3:** Average aerodynamic forces during the upstroke, downstroke, and full flapping cycle for PDMS wing membranes with different A:B ratios.

		${\bar{F}}_{V}\;(N)$			${\bar{F}}_{H}\;(N)$		
		Downstroke	Upstroke	Average	Downstroke	Upstroke	Average
10:1		0.1362	−0.0072	0.0645	0.3665	−0.1885	0.089
30:1		0.1406	0.0019	0.0713	0.3823	−0.192	0.0951
50:1		0.1494	−0.0042	0.0726	0.3293	−0.1778	0.0757

## Data Availability

The data supporting this study’s findings are available from the corresponding author upon reasonable request.
